# Gastrointestinal stromal tumor of the rectum with scapular metastasis: a case report

**DOI:** 10.1186/1752-1947-6-145

**Published:** 2012-06-07

**Authors:** Fatih Selcukbiricik, Deniz Tural, Mehmet Akif Ozturk, Sergulen Dervisoglu, Sait Sager, Murat Hız, Nil Molinas Mandel

**Affiliations:** 1 Department of Clinical Oncology, University of Istanbul, Cerrahpasa School of Medicine, 34098, Cerrahpasa Fatih, Istanbul, Turkey; 2 Department of Pathology, University of Istanbul, Cerrahpasa School of Medicine, 34098, Cerrahpasa Fatih, Istanbul, Turkey; 3 Department of Nuclear Medicine, University of Istanbul, Cerrahpasa School of Medicine, 34098, Cerrahpasa Fatih, Istanbul, Turkey; 4 Department of Orthopedics, University of Istanbul, Cerrahpasa School of Medicine, 34098, Cerrahpasa Fatih, Istanbul, Turkey

## Abstract

****Introduction**:**

Gastrointestinal stromal tumors are rare tumors. They commonly metastasize within the abdominal cavity, particularly to the liver. Less commonly, metastases can be found in the bone.

****Case presentation**:**

We here present a case of metastasis to the scapula in a 54-year-old Caucasian male patient with an advanced gastrointestinal stromal tumor, which was subsequently successfully treated with resection and sunitinib.

****Conclusion**:**

The present study is, to the best of our knowledge, the second to describe scapular metastasis of a gastrointestinal stromal tumor. Our patient was treated by scapulectomy. The overwhelming majority of scapular tumors are metastases that arise from soft tissue, hepatocellular and thyroid tumors. Gastrointestinal stromal tumor metastasis occurs rarely. Scapular surgery can successfully provide local control of the disease. After the surgery, patients should continue with medical treatment.

## **Introduction**

Gastrointestinal stromal tumors (GISTs) are the most common connective tissue tumors of the gastrointestinal (GI) tract [1]. GISTs originate from the interstitial cells of Cajal [2]. These cells are a type of interstitial cell found in the GI tract and are involved in the generation of electrical pacemaker activity for GI motility. The majority of GISTs are associated with activating mutations in the KIT gene or platelet-derived growth factor receptor alpha (PDGFRα) [3]. Ten to twenty patients in every one million have a new diagnosis of GIST per year in the US and 5,000 to 6,000 cases are diagnosed every year. In Europe, the incidence is estimated to be 6.6 to 14.5 cases in every 3 million to 7 million people [4,5].

Surgery is the standard treatment for primary resectable GISTs; however, surgical resection is seldom curative and by five years after complete removal, 40% of patients have relapsed [6]. Imatinib is the first-line treatment in patients with metastatic or unresectable GISTs. Its use results in durable objective response or stable disease in approximately 85% of patients with advanced GISTs, and it is well tolerated [7,8]. Sunitinib is the recommended option for documented GISTs (resectable, unresectable, recurrent or metastatic disease) in patients that experience life-threatening side effects from imatinib therapy, and as a treatment for unresectable or metastatic disease following unsuccessful imatinib treatment [9].

## **Case presentation**

A 54-year-old Caucasian male patient with no previous history of any disease was referred to our clinic on January 2001 with rectal bleeding. A colonoscopy showed an ulcerated polypoid mass in his rectum that was 12 cm distant from the anal verge. A pathological examination of the biopsied specimens revealed a GIST. Surgery was performed and the mass was excised by *en bloc* resection technique. A computed tomography (CT) scan of his thorax demonstrated no pathological finding. Multiple and non-resectable bilobar liver metastases were found on abdominal magnetic resonance imaging (MRI). Our patient was started on imatinib 400 mg daily in 2001. After an event-free follow-up period, our patient was readmitted in 2010 with complaints of right shoulder pain and limitation of shoulder movement. His liver metastases were seen to be stable on the abdominal and pelvic MRI. No local recurrence was found during a colonoscopic examination.

**Figure 1:**
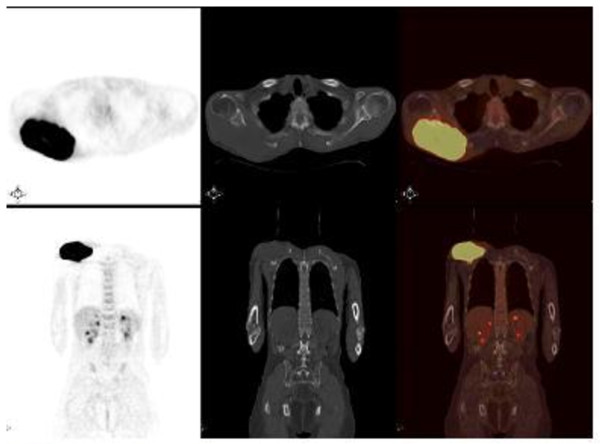
**Fluorodeoxyglucose positron emission tomography-computed tomography.** The patient was intravenously injected 455 MBq (12.3 mCi) of F-18 fluorodeoxyglucose after 6 hours of fasting. After one hour of waiting time in a silent room, the patient was imaged using an integrated positron emission tomography-computed tomography camera, which consists of a six-slice computed tomography gantry integrated on a lutetium oxyorthosilicate-based fullring positron emission tomography scanner (Siemens Biograph 6, IL, USA). **(A)** Anterior-posterior maximum intensity projection positron emission tomography image; **(B)** axial positron emission tomography and **(D)** axial fusion images show intense hypermetabolic mass with a maximum standard uptake value (SUVmax) of 15.2 at the level of infraspinatus muscle in the right posterior shoulder. **(C)** Axial computed tomography image shows a soft tissue mass destructing the right scapula in the right posterior shoulder. Maximum intensity projection image shows another focus of fluorodeoxyglucose uptake in the midline of the upper pelvis.

**Figure 2:**
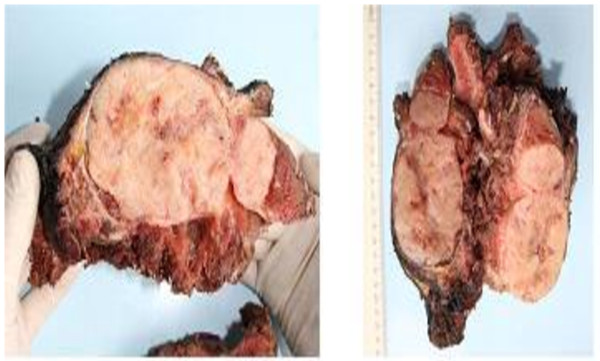
**Material resected from the scapula and peripheral tissue mass.** An elastic nodular mass is observed on the anterior (outer, ventral) surface of the scapula, 13 cm × 10 cm × 5 cm in size, with a 0.2 cm-thick pseudocapsule with a grayish yellow cut surface. The mass destructed the medullary bone medially 6 cm in length and shows soft tissue invasion. Soft tissue distance to surgical border is 2 cm.

**Figure 3:**
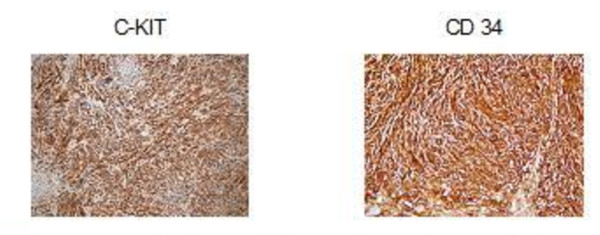
**An eosinophilic tumor with lucid vacuolar cytoplasm, reminiscent of smooth muscle and composed of interlacing bundles of fusiform cells was observed.** The immune profile of the metastatic tumor (c- kit and CD34 positive) and previous biopsy together confirmed the diagnosis of a GIST.

MRI showed a 13 cm × 10 cm × 7 cm mass with partial contrast-enhancement that was T1- and T2-hyperintense and heterogeneous. The mass was destroying the body of his right scapula and had widespread involvement in the infraspinatus muscle. A fluorodeoxyglucose positron emission tomography (PET)-CT showed a hypermetabolic mass in his posterior right shoulder (Figure 1). The mass was resected (Figure 2). On microscopy, the tumor was composed of an interlacing pattern of spindle cells that stained negative for smooth muscle actin, and positive for CD117 (c-kit) and CD34 (Figure 3). Pathologic findings were compatible with metastasis of GIST to the scapula. Following resection, his imatinib therapy was changed to sunitinib. At the time this report was written, our patient was still taking sunitinib 50 mg daily.

## **Discussion**

GISTs are mesenchymal tumors with generic histological features that arise from the gastrointestinal or abdominal tract. GISTs originate from interstitial cells of Cajal - intestinal pacemaker cells that arise from the muscularis propria of the gastrointestinal tract wall [10]. The *c-kit* gene encodes an oncogenic transmembrane receptor tyrosine kinase (KIT), whose ligand is a stem cell factor. The *PDGFRα* gene also encodes an oncogenic transmembrane receptor tyrosine kinase (PDGFRα).

Gain of function mutations of the *c-kit* and *PDGFRα* genes are associated with the tumorigenesis of GISTs. The most frequent mutations are observed in the juxtamembrane domain of KIT, which is coded by exon 11. Mutations in the extracellular domain of KIT exon 9 are seen less often. Although most GISTs express KIT (95%), a minority will be negative for KIT or express wild-type KIT [11]; however, in some GISTs, a mutation cannot be detected. The epidemiology of GISTs is not fully known; on average, 10 cases per million individuals are thought to occur. Most patients are aged 40 to 80 years old and are diagnosed at a median age of 60 years [12].

GISTs commonly occur in the stomach (50% to 60%) and small intestine (25%). They can also occur in the colorectum (10%), esophagus (5%), anywhere along the GI tract and, rarely, in extraintestinal sites, including the mesentery, omentum, peritoneum, gallbladder and liver (3%) [13]. The clinical presentation of GISTs is largely dependent on size. Small tumors (≤2 cm) usually do not produce symptoms and are often detected incidentally via endoscopy or radiographic examination. Several major symptoms, which are not only GIST-related, include bleeding, upper abdominal pain, fullness and abdominal mass and obstruction [14]. Sometimes, urgent abdominal complaints, such as abdominal bleeding, massive gastrointestinal bleeding, perforation or obstruction, may occur.

Biopsy material is essential for the definitive diagnosis of GISTs, as radiological and endoscopic examinations are insufficient as diagnostic tools. A percutaneous biopsy should not be performed in patients with potentially resectable tumors, due to the low diagnostic yield and the risk of seeding malignant cells. GISTs present in a wide range of size (from a few millimeters to >30 cm). They commonly arise from the wall of the GI tract. GISTs are usually well-demarcated encapsulated firm nodules with a tan-gray appearance on the cut surface. Large tumors may show cystic degeneration, central necrosis and hemorrhage. GISTs can be composed of spindle cells, epithelioid cells, or a mixture of both spindle and epithelioid cells (pleomorphic). The best immunostaining method for identifying GISTs is to test for expression of KIT, also known as CD117. About 5% of GISTs do not stain positive for KIT. Other immunohistochemical markers often observed to be positive in GISTs are CD34 (60% to 70%) and smooth muscle actin (30% to 40%). A small percentage of GISTs may show positivity for desmin, or S-100. This is not important if the GIST is *c-kit*-positive, several immunohistochemical markers in the diagnosis of GISTs, the differential diagnosis and prognosis assessment is currently under investigation [15,16].

The liver is the most common site of GIST metastasis. Distant metastasis to other sites, especially bone, the lungs and lymph nodes, is relatively rare. The mandible, spine, femur and humerus have been reported as localizations of bone metastasis. Only one case of scapular metastasis has been reported [17]. The presented report is the second to describe scapular metastasis. Our patient presented with a right shoulder mass. GIST metastasis was confirmed via biopsy and our patient underwent scapulectomy. The overwhelming majority of scapular tumors are metastases from soft tissue, hepatocellular and thyroid tumors. GIST metastases are rare. Scapula surgery can successfully provide local control of the disease. Following such surgery, patients should continue with medical treatment.

According to a retrospective analysis, 17 of 309 GIST patients (5.5%) had bone metastasis; scapular metastasis was not observed and bone metastasis was not a primary clinical feature at their initial presentation. The patients with bone metastasis had a median survival of 325 weeks from initial diagnosis, 252 weeks from distant recurrence-free status, and 135 weeks after the diagnosis of bone metastasis. CT, MRI and PET-CT were used to identify metastases; however, PET-CT was more effective for diagnosis, staging, restaging and measurement of therapeutic response of these neoplasms. Bone scanning was not routinely performed [18].

Despite advances in drug therapy, surgical resection remains the primary treatment of GISTs and total resection remains the most effective. However, drug treatment should be used instead of surgery in some patients, such as those with widespread metastasis, local progression or a general medical condition that contraindicates surgery, and when the surgical intervention is associated with a high rate of morbidity and/or mortality. If technically feasible, resection can be performed in patients with liver metastasis; however, surgery is often not applicable due to multiple liver metastases, very large metastases or peritoneal involvement [19]. Hepatic artery embolization or chemoembolization is thought to be an effective palliative treatment because GISTs are often hypervascular.

Imatinib mesilate inhibits KIT tyrosine kinase activity. Imatinib is the first molecular targeted therapy. The current recommendation is to initiate imatinib treatment at 400 mg daily for at least three months and then commence follow-up. Initiation at 800 mg daily often causes side effects, which eventually lead to dose reduction. Thus, an initial dose of 400 mg daily can be increased according to follow-up results [20,21]. In patients that do not respond to high-dose imatinib or if resistance develops over time, imatinib must be substituted with sunitinib. Sunitinib malate does not cure cancer like imatinib does, but it is the generally accepted second-line treatment, as sunitinib prolongs survival in patients with advanced GISTs [22].

## **Conclusion**

The present study is the second to describe scapular metastasis of a GIST. Our patient was treated by scapulectomy. The overwhelming majority of scapular tumors are metastases that arise from soft tissue, hepatocellular and thyroid tumors. GIST metastasis occurs rarely. Scapular surgery can successfully provide local control of the disease. Following the surgery, patients should continue with medical treatment.

## **Consent**

Written informed consent was obtained from the patient for publication of this manuscript and any accompanying images. A copy of the written consent is available for review by the Editor-in-Chief of this journal.

## **Competing interests**

The authors declare that they have no competing interests.

## **Authors’ contributions**

All the authors have contributed to the submitted case report. The design of the case report was done by all authors. Imaging and pathology studies were realized by SD, MH and SD. Analysis and interpretation of the data were realized by DT, FS, MAÖ and NMM. The results were approved by all authors. All authors read and approved the final manuscript.
